# Structure-guided deep learning for back acupoint localization via bone-measuring constraints

**DOI:** 10.3389/fphys.2025.1662104

**Published:** 2025-08-26

**Authors:** Yulong Wang, Tian Lan, Wenjian Dou, Zhi Chen, Song Zhang, Gong Chen

**Affiliations:** ^1^ School of Artificial Intelligence and Information Technology, Nanjing University of Chinese Medicine, Nanjing, China; ^2^ School of Computer Science, Inner Mongolia University, Hohhot, China; ^3^ Affiliated Hospital of Nanjing University of Chinese Medicine, Nanjing, China; ^4^ Institute of Chinese Medicine Literature, Nanjing University of Chinese Medicine, Nanjing, China

**Keywords:** acupoint localization, HRFormer, anatomical landmark detection, bone-measuring method, medical imaging, artificial intelligence

## Abstract

Accurate acupoint localization is crucial for the effectiveness of acupuncture and related Traditional Chinese Medicine (TCM) therapies. This study introduces a novel automated framework for recognizing back acupoints, uniquely integrating the traditional TCM bone-measuring principle with advanced deep learning for medical image analysis. The method employs an HRFormer backbone network combined with a Structure-Guided Keypoint Estimation Module (SG-KEM) and a structure-constrained loss function, ensuring anatomically consistent predictions within a standardized spatial coordinate system to improve accuracy across diverse body types. Trained and evaluated on a dataset of 430 high-resolution back images with 19 annotated acupoints, the framework achieved a normalized mean error (NME) of 0.6%, a failure rate (FR@1 cm) of 1.2%, an area under the curve (AUC) of 0.97, and a precision of 93.8%, while operating in real-time at 18 frames per second. Component analysis confirmed significant contributions: the SG-KEM module reduced the mean error by 33.3%, and the structure-constrained loss further decreased it to 0.6%. Moreover, ablation studies under challenging conditions validated the model’s robustness. On the obese subset, the NME decreased from 1.5% to 0.8%, FR@1 cm dropped from 4.0% to 1.3%, and precision improved from 83.8% to 93.4%. Under illumination variation, the model achieved an NME of 0.9%, outperforming both HRFormer (1.3%) and HRFormer+SG-KEM (1.1%), with corresponding increases in AUC and precision. These findings demonstrate strong generalization across diverse clinical scenarios. Collectively, these results establish a clinically viable and computationally efficient solution for intelligent acupoint localization, supporting AI-assisted diagnosis and personalized treatment strategies within modern TCM healthcare systems.

## 1 Introduction

Traditional Chinese Medicine (TCM) is a comprehensive medical system with a history spanning thousands of years and has gained widespread application worldwide through extensive clinical practice (Fung, 2009). Rooted in the theories of zang-fu organs and meridians, TCM prominently features acupuncture and massage, which are primarily utilized for disease prevention, treatment, and alleviation of fatigue (Epstein et al., 2023). Acupuncture and massage achieve therapeutic effects and fatigue relief by stimulating specific acupoints on the human body, thereby regulating the flow of qi and blood and achieving a balance of yin and yang (Ma, 2021; Cai and Vasconcelos, 2018). Accurate acupoint localization is critical to the efficacy of acupuncture and massage therapies, as inaccuracies can directly affect treatment outcomes. Historically, acupoint identification has often relied on clinical experience, potentially leading to inconsistent treatment effects. Therefore, the development of high-precision acupoint recognition technologies holds significant promise for enhancing acupuncture accuracy, facilitating the modernization of TCM, and promoting intelligent diagnostic systems (Qi et al., 2024; Jaladat et al., 2023; Shen et al., 2024).

Furthermore, recent studies have demonstrated that accurate stimulation of specific acupoints can modulate cortical activity and brain network connectivity, revealing the neurophysiological basis of acupuncture efficacy. EEG- and fMRI-based evidence shows that acupuncture at well-localized points can regulate spectral power and functional connectivity in the brain, contributing to therapeutic effects in neurological conditions such as epilepsy and Parkinson’s disease (Xue et al., 2023; Yu et al., 2018; Yu et al., 2019; Yu et al., 2024). In particular, decoding brain responses to acupuncture using EEG representation learning has laid the foundation for intelligent acupuncture–brain interfaces (Lei et al., 2023; Yu et al., 2025). These findings highlight the critical need for precise and automated acupoint localization as a prerequisite for advancing brain-targeted TCM therapies and integrative medicine systems.

In recent years, deep learning has increasingly found applications in the medical field, offering promising opportunities for the modernization of TCM (Pan et al., 2024; Zhou et al., 2024). Yang et al. proposed a deep learning-based method for back acupoint localization on weak-feature body surfaces, incorporating attention mechanisms to enhance feature representation (Yang et al., 2024). However, their method primarily relies on pixel-level intensity and lacks anatomical structure modeling, which limits its ability to adapt to individual variations in body shape or posture. Moreover, it does not introduce normalization mechanisms like bone-based coordinate systems, which are essential for physiologically consistent keypoint detection (Zhang et al., 2024). Our method explicitly addresses these challenges by incorporating a structure-guided estimation module and a TCM-inspired bone-measuring loss function, enabling better generalization and anatomical fidelity. Researchers such as Alexopoulos et al. (2023) have used deep learning for the early detection of knee osteoarthritis, while Panda et al. (2024) have applied it to lung tissue classification. Ronneberger et al. (2015) Unet model has become a staple in biomedical image segmentation, and Lee et al. (2018) have developed convolutional neural network models for the classification of dental diseases.

Several studies have showcased the potential of deep learning in acupoint recognition (Li et al., 2024). Sun et al. (2022) focused on auricular point localization by constructing a 91-keypoint dataset and applying directional normalization modules, achieving high precision in ear-based acupuncture. Wang et al. (2023) Hand acupuncture point localization method based on a dual-attention mechanism and cascade network model to localize 21 hand acupoints with excellent real-time performance. Similarly, Yuan et al. (2024) proposed the YOLOv8-ACU framework for facial acupoint detection, incorporating lightweight ECA modules and a Slimneck structure to balance model accuracy and efficiency. While these approaches demonstrate strong performance in their respective body regions, they primarily target areas with rich local features and fixed landmarks, such as the ears, hands, and face. In contrast, the human back lacks visually salient landmarks and exhibits considerable variation in body morphology, making acupoint localization significantly more challenging (Yang et al., 2024).

While these methods offer unique advantages in their respective body regions, research focusing on back acupoints remains limited. Compared to acupoint areas like the ear or face, back acupoint recognition presents distinct challenges due to the lack of clear reference structures, a generally flat surface, and indistinct landmarks, posing significant challenges for automated localization (Kim et al., 2023; Mao et al., 2021). Nevertheless, the back hosts numerous vital back-shu points closely linked to internal organ functions, bearing irreplaceable clinical significance (Kim et al., 2023). Therefore, improving back acupoint recognition accuracy is critical for advancing intelligent diagnosis in TCM.

To overcome these limitations, we propose a novel acupoint detection method that leverages both structural and contextual knowledge. Building upon HRFormer (Yuan Y. et al., 2021), a high-resolution transformer network for dense prediction tasks, we introduce the Structure-Guided Keypoint Estimation Module (SG-KEM) to explicitly integrate osteological priors from Traditional Chinese Medicine. In addition, we design a structure-constrained loss based on bone-proportion theory (Gang et al., 2011), enabling the model to predict acupoints within a normalized anatomical coordinate system. Unlike prior methods, our approach accounts for both pixel-wise accuracy and physiological consistency, achieving high precision while maintaining robustness across individuals with varying body proportions and imaging conditions.

## 2 Materials and methods

### 2.1 Dataset

To support model training and evaluation, we utilized the publicly available DMD-BAK dataset, which addresses the lack of large-scale annotation resources for back acupoint localization. The dataset is accessible at https://www.kaggle.com/datasets/chunzheye/dmd-bak and contains 2,691 high-resolution JPG images of the human back. Professional Traditional Chinese Medicine (TCM) practitioners were invited to assist in both the selection of 430 representative images—based on pose diversity, image clarity, and annotation completeness—and the re-annotation of acupoint positions to ensure accuracy and clinical validity. In addition, new annotation modules were incorporated to enrich the dataset structure and facilitate subsequent model training. Each selected image includes standardized annotations for 19 back acupoints. All data remain anonymized and ethically compliant. The final subset offers a practical balance between anatomical diversity and computational feasibility, making it well suited for deep learning-based localization tasks. Table 1 lists the corresponding acupoint codes and names, and Table 2 summarizes acupoint–skeletal correlations with topologic descriptors.

**TABLE 1:** The correspondence between the code numbers and the acupoints.

Seq.	Type	Code	Acupoint	Index
1	DU	DU14	Dazhui	1
2	BL	BL11	Dazhu	2/3
3	BL	BL12	Fengmen	4/5
4	BL	BL13	Feishu	6/7
5	BL	BL14	Jueyinshu	8/9
6	BL	BL15	Xinshu	10/11
7	BL	BL17	Geshu	12/13
8	BL	BL18	Ganshu	14/15
9	BL	BL19	Danshu	16/17
10	BL	BL20	Pishu	18/19
11	BL	BL21	Weishu	20/21
12	BL	BL22	Sanjiaoshu	22/23
13	BL	BL23	Shenshu	24/25
14	BL	BL25	Dachangshu	26/27
15	BL	BL43	Gaohuang	28/29
16	GB	GB21	Jianjing	30/31
17	SI	SI9	Jianzhen	32/33
18	SI	SI10	Naoshu	34/35
19	SI	SI11	Tianzong	36/37

**TABLE 2:** Acupoint-skeletal correlations with topologic descriptors.

Seq.	Acupoint	Skeletal landmark	Topological relationship
1	Dazhui	C7 spinous process	Below C7 (midline)
2	Dazhu	T1 Spinous Process	Below T1, 1.5 cun lateral
3	Fengmen	T2 Spinous Process	Below T2, 1.5 cun lateral
4	Feishu	T3 Spinous Process	Below T3, 1.5 cun lateral
5	Jueyinshu	T4 Spinous Process	Below T4, 1.5 cun lateral
6	Xinshu	T5 Spinous Process	Below T5, 1.5 cun lateral
7	Geshu	T7 Spinous Process	Below T7, 1.5 cun lateral (scapula level)
8	Ganshu	T9 Spinous Process	Below T9, 1.5 cun lateral
9	Danshu	T10 Spinous Process	Below T10, 1.5 cun lateral
10	Pishu	T11 Spinous Process	Below T11, 1.5 cun lateral
11	Weishu	T12 Spinous Process	Below T12, 1.5 cun lateral
12	Sanjiaoshu	L1 Spinous Process	Below L1, 1.5 cun lateral
13	Shenshu	L2 Spinous Process	Below L2, 1.5 cun lateral (umbilicus level)
14	Dachangshu	L4 Spinous Process	Below L4, 1.5 cun lateral (iliac crest level)
15	Gaohuang	T4 Spinous Process	Below T4, 3 cun lateral (medial scapula)
16	Jianjing	C7 and Acromion	Midpoint of C7-Acromion line
17	Jianzhen	Posterior Axillary Fold	1 cun above axillary fold
18	Naoshu	Scapular Spine	Inferior to scapular spine
19	Tianzong	Scapular Spine	Center of infraspinous fossa

To ensure robust model evaluation, the dataset was divided into training, validation, and testing subsets at a 6:2:2 ratio. The validation set was strictly separated from the training data to prevent data leakage, allowing for accurate monitoring of generalization performance and effective hyperparameter tuning.

### 2.2 Data preprocessing

Given the relatively limited dataset size, multiple data augmentation techniques were applied exclusively to the training set to enhance generalization and model robustness. These included random horizontal flipping (p = 0.5), affine transformations (rotation within ±15°, scaling between 0.9 and 1.1), and adjustments in brightness and contrast (scaling factors between 0.8 and 1.2). All augmented samples retained label consistency (Wu et al., 2022). The test set remained unaltered to ensure fair evaluation (Table 3). summarizes the augmentation strategies.

**TABLE 3:** Data augmentation strategies.

Augmentation type	Description	Parameter range/Probability
Random Horizontal Flip	Symmetric left-right flip of the image	Probability = 0.5
Random Rotation	Rotation operation in affine transformation	Angle ∈ [−15°, +15°]
Random Scaling	Affine scaling operation with the center unchanged	Scaling ratio ∈ [0.9, 1.1]
Random Brightness Perturbation	Adjust the overall brightness of the image	Adjustment factor ∈ [0.8, 1.2]
Random Contrast Perturbation	Change the contrast of the image	Adjustment factor ∈ [0.8, 1.2]

Following data cleansing and augmentation, the final dataset included 258 training images, 86 validation images, and 86 testing images, all manually verified for clarity and annotation accuracy.

### 2.3 Backbone network

**FIGURE 1:**
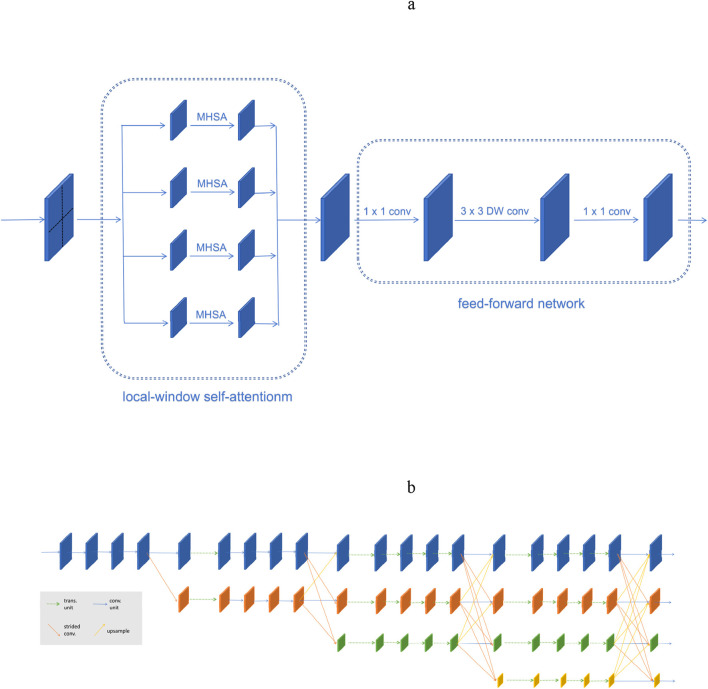
Structure of HRFormer. **(a)**The HRFormer block is composed of local-window self-attentionm and feed-forward network (FFN) with depth-wise convolution. **(b)**Illustrating the HRFormer architecture.

The HRFormer architecture was adopted as the backbone of our model due to its superior performance in dense prediction tasks. HRFormer integrates the multi-resolution parallel structure of HRNet with the global modeling capability of Transformers. The network comprises four stages (Stage 1 to Stage 4), each containing multiple branches of varying resolutions. Transformer blocks within each stage operate in windowed self-attention mode, and feature maps are fused across branches to preserve both fine-grained spatial information and high-level semantic understanding.

(Table 4) outlines the structural configuration of HRFormer. Maintaining a high-resolution stream throughout the network enables the precise representation of acupoint features. Furthermore, the network’s capacity to process skeletal structures (e.g., spinal curvature, scapular positions) alongside fine local details (e.g., inter-acupoint distances) enhances both accuracy and generalization. The architecture is illustrated in (Figure 1).

**TABLE 4:** Parameters of four stages of the HRFormer.

Stage	Number of branches	Resolution ratio	Number of transformer blocks per branch	Embedding dimension per layer(C)
1	1	1/4	2	64
2	2	1/4,1/8	2,2	64,128
3	3	1/4,1/8,1/16	2,2,2	64,128,256
4	4	1/4,1/8,1/16,1/32	2,2,2,2	64,128,256,512

### 2.4 The SG-KEM module

**FIGURE 2:**
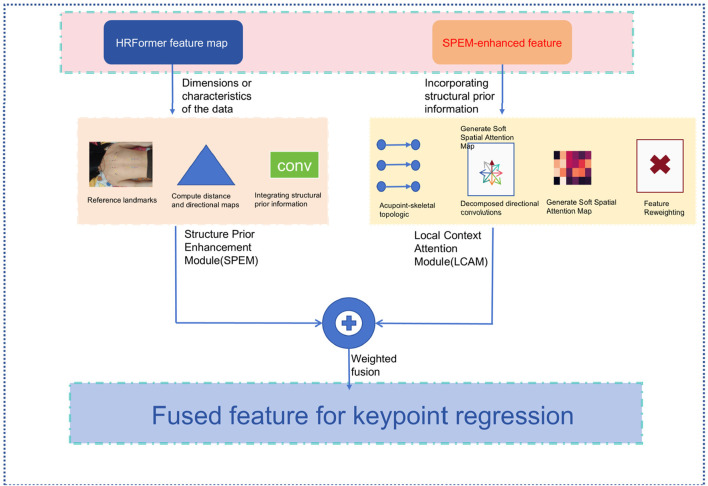
Structure of SG-KEM module.

To address the challenge of low visual salience in back acupoint recognition, we propose the Structure-Guided Keypoint Estimation Module (SG-KEM). This module is designed to integrate prior knowledge of human skeletal structures with context-aware features from the neighborhoods of keypoints, guiding the model to focus on anatomically meaningful regions and thereby improving the robustness and accuracy of keypoint localization. SG-KEM consists of two submodules: the Structural Prior Enhancement Module (SPEM), which models the relationship between acupoints and skeletal structures to provide structural guidance; and the Local Context Attention Module (LCAM), which enhances semantic representation in local regions through a lightweight attention mechanism. These two components work synergistically to improve the model’s ability to adapt to complex backgrounds and individual variations (Figure 2).

#### 2.4.1 Structure prior enhancement module (SPEM)

SPEM introduces anatomical constraints derived from Traditional Chinese Medicine (TCM) knowledge by leveraging a set of bone-referenced landmarks $B={\left\{b_{k}\right\}}_{\left\{k=1\right\},}^{k}$ such as the seventh cervical vertebra and inferior scapular angles. These landmarks are manually annotated and exhibit stable relative positions across individuals.

We construct a set of geometrical edges Equation 1:

$$E=\{\left(i,j\right)|b_{i},b_{j}\in B,D_{ij}^{\text{ref}}\;\left.known\right\}$$ (1)

For each pair $\left.\left(i,j\right)\in E\right)$ , we compute a distance map $\left(D_{\text{ij}}\right)$ and a directional map $\left(A_{\text{ij}}\right)$ based on predicted keypoint locations $\left(p_{i}p_{j}\right)$ Equation 2.

$$D_{ij}\left(x,y\right)\left.=\right|p_{i}-p_{j}{|}_{2},A_{ij}\left(x,y\right)=\frac{p_{i}-p_{j}}{\left|p_{i}-p_{j}{|}_{2}\right.}$$ (2)

These are concatenated to form a structural guidance tensor $G_{\text{prior}}$ , which is fused with the HRFormer feature map $F\text{ via }a\;\left(1\times 1\right)$ convolution Equation 3:

$$F_{SPEM}=F+{Conv}_{1\times 1}\left({Concat}_{\left(i,j\right)\in E}\left[D_{ij},A_{ij}\right]\right)$$ (3)

This fusion enables the network to be aware of physiologically plausible acupoint arrangements.

#### 2.4.2 Local contextual attention module (LCAM)

To model local dependencies and eliminate background noise, LCAM applies directional convolution and spatial attention. The SPEM-enhanced feature $F_{\text{SPEM}}$ is first processed with decomposed directional convolutions Equation 4:

$$F_{dir}=ReLU\left({Conv}_{3\times 1}\left(F_{LCAM}\right)+{Conv}_{1\times 3}\left(F_{SPEM}\right)\right)$$ (4)

Next, we generate a soft spatial attention map Equation 5:

$$A_{\left(x,y\right)}=\frac{exp\left(Q_{\left(x,y\right)}\right)}{\sum_{\left(u,v\right)}\;exp\left(Q_{\left(u,v\right)}\right)},Q={Conv}_{1\times 1}F_{dir}$$ (5)

The final output is a reweighted feature map Equation 6:

$$F_{LCAM}=F_{dir}⊙A$$ (6)

#### 2.4.3 Output fusion

We fuse the outputs of SPEM and LCAM to obtain the final structurally enhanced feature representation for keypoint regression Equation 7:

$$F_{SG-KEM}=\left({Conv}_{3\times 3}\left(F_{SPEM}{\;+\;F}_{LCAM}\right)\right)$$ (7)

This fused representation is passed to the keypoint regression head for heatmap generation.

### 2.5 Structure-constrained loss function

To fully leverage the structure-guided features extracted by the SG-KEM module, the resulting feature map is used as the output of the keypoint heatmap branch to regress the spatial coordinates of acupoints. To enhance both localization accuracy and anatomical plausibility, we design a structure-constrained loss function based on the traditional Chinese medicine (TCM) bone-measuring method.

Unlike general pose estimation tasks involving full-body joint detection, our method only focuses on stable anatomical landmarks in the back region relevant to acupoint localization. These landmarks obtained through a lightweight anatomical landmark detection module. Based on these reference points, we divide the trunk region into standardized proportional units known as “cuns” using the TCM bone-measuring method. This process constructs a subject-specific proportional reference space, onto which all acupoint annotations are projected based on proportional units (cun).

**FIGURE 3:**
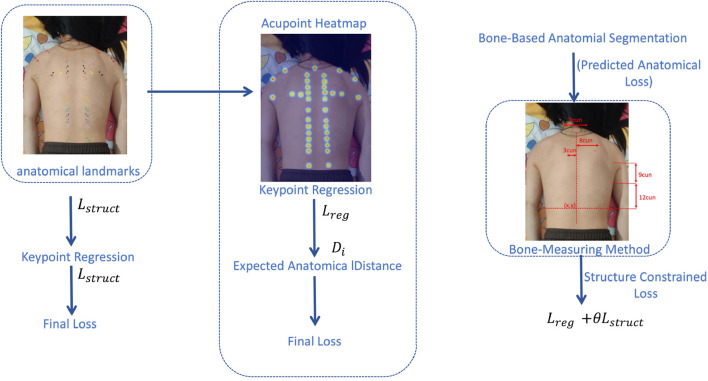
Structure-constrained Losses function.

The normalized coordinate space eliminates differences in body proportion and posture, allowing the model to learn acupoint localization in a structurally consistent and interpretable manner. The architectural framework of this method is shown in (Figure 3).

To incorporate anatomical structure into the training process, we introduce a sample-specific normalization mechanism. Specifically, we estimate a personalized scale factor r , representing the number of pixels per cun for each sample. This factor is computed using the Euclidean distance d between two stable anatomical landmarks—typically the medial borders of the left and right scapulae—defined to span 24 cuns according to TCM standards Equation 8:

$$r=\frac{d}{24}$$ (8)

This normalization enables the model to apply structural constraints in a physiologically consistent coordinate space, allowing adaptation to individuals with varying body proportions while preserving standard acupoint relationships.

To encode structural knowledge, we define a set of acupoint pairs P with known anatomical distances derived from TCM literature. These include bilateral Dachangshu (BL25), Xinshu (BL15), and spinal-axis acupoints such as Dazhui (DU14). For each acupoint pair, the expected distance in cuns $d_{\text{cun}}$ is translated into pixel space using the personalized scale α .

According to TCM standards, the distance between specific acupoint pairs (e.g., bilateral BL25) is defined as a fixed number of cuns (e.g., six cuns), which is then converted to pixel distance by multiplying with the scale factor α Equation 9:

$$d_{pix}^{expected}=\alpha \cdot d_{cun}$$ (9)

This step ensures anatomical distances are expressed in the same coordinate system as the predicted keypoint locations, enabling consistent comparison during optimization.

The structure-constrained loss is formulated as a mean squared error between the predicted distance and the expected anatomical distance Equation 10:

$$L_{struct}=\frac{1}{M}{\sum_{\left(i,j\right)\in P}\left({\left\|{\hat{p}}_{i}-{\hat{p}}_{j}\right\|}_{2}-r\cdot l_{ij}\right)}^{2}$$ (10)

Here, P denotes the set of acupoint pairs selected for structural constraint, and M is the total number of constrained pairs.

To jointly optimize both pixel-level accuracy and anatomical consistency, we define the final loss function as a weighted sum of keypoint regression loss $L_{\text{reg}}$ and the structural loss $L_{\text{struct}}$ Equation 11:

$$L_{total}=L_{reg}+\theta \cdot L_{struct}$$ (11)

Where θ is a hyperparameter balancing precision and structural compliance.

### 2.6 Evaluation metrics

To evaluate the performance of the proposed model, we adopted five standard metrics: Normalized Mean Error (NME) (Lai et al., 2019), Failure Rate (FR) (Finkelstein, 2008), Area Under the Curve (AUC) (Myerson et al., 2001), Precision (Streiner and Norman, 2006), and Frames Per Second (Image/s) (Koslowsky et al., 2006). These metrics jointly assess the model’s accuracy, robustness, and efficiency.

Normalized Mean Error (NME): The average Euclidean distance between predicted and ground-truth keypoints, normalized by inter-Xinshu distance Equation 12:

$$NME=\frac{1}{K\cdot N}\sum_{k=1}^{K}\sum_{n=1}^{N}\frac{\left\|Y_{k}^{n}-{\hat{Y}}_{k}^{n}\right\|}{d}\times 100\%$$ (12)

Failure Rate (FR): The proportion of test samples with NME exceeding a fixed threshold δ Equation 13:

$$FR=\frac{1}{K}\sum_{k=1}^{K}I\left(\frac{1}{N}\sum_{n=1}^{N}\frac{\left\|Y_{k}^{n}-{\hat{Y}}_{k}^{n}\right\|}{d}>\delta \right)\times 100\%$$ (13)

Area Under the Curve (AUC): The integral of the cumulative error distribution curve from 0 to δ Equation 14:

$$AUC=\int_{0}^{10\%}CED\left(\delta \right)\;d\delta$$ (14)

Precision: The proportion of true positive predictions among all positive predictions Equation 15:

$$Precision=\frac{TP}{TP+FP}$$ (15)

Images Per Second (IPS): The number of Images per second Equation 16:

$$IPS=\frac{Images}{s}$$ (16)

## 3 Result

The proposed model was trained and evaluated in a high-performance computing environment equipped with an NVIDIA GeForce RTX 3080 Ti GPU, running Ubuntu 18.04. The implementation was based on Python 3.8 and PyTorch 1.10. The Adam optimizer was used, with a batch size of 32 and a dropout rate of 0.5 to mitigate overfitting. Training was conducted over 100 epochs with early stopping based on validation performance. These configurations ensured training efficiency and model generalization.

### 3.1 Performance comparison with baseline models

**FIGURE 4:**
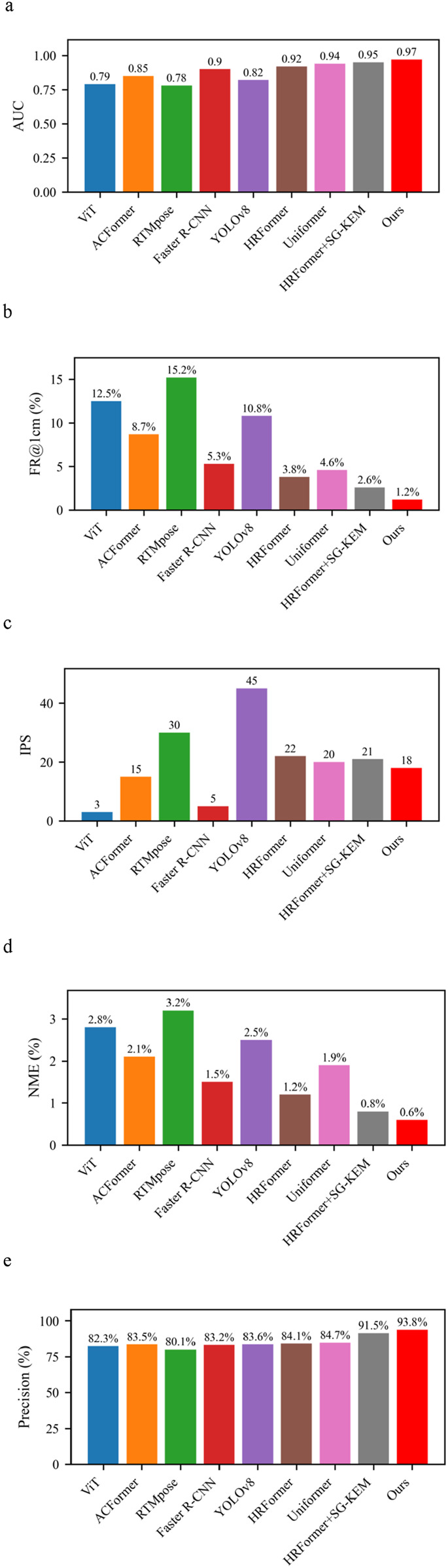
Performance comparison of different models. **(a)** Comparison of different models in AUC. **(b)** Comparison of different models in FR@1 cm (%). **(c)** Comparison of different models in IPS. **(d)** Comparison of different models in NME (%). **(e)** Comparison of different models in Precision (%).

To evaluate the effectiveness of the proposed method, we conducted comparative experiments against several mainstream models, including Vision Transformer (ViT) (Yuan L. et al., 2021)., ACFormer (Zong et al., 2023), RTMpose (Jiang et al., 2023; He et al., 2024), Faster R-CNN(Bharati and Pramanik, 2019), YOLOv8 (Sohan et al., 2024),HRFormer (Yuan Y. et al., 2021) and Uniformer (Li et al., 2023). The comparison focused on five key metrics: Normalized Mean Error (NME), Failure Rate within 1 cm (FR@1 cm), Area Under the Curve (AUC), Precision, and Images Per Second (IPS). The results are presented in (Figure 4).

The Normalized Mean Error (NME) dropped significantly to 0.6%, representing a 57.1% reduction compared to ViT (2.8%) and a 50% reduction compared to HRFormer (1.2%). Similarly, the FR@1 cm metric improved markedly, reaching only 1.2%, versus 12.5% for ViT and 3.8% for HRFormer, indicating high localization precision at the anatomical level.

In terms of AUC, the proposed method achieved 0.97, outperforming all others, including Uniformer (0.94) and HRFormer (0.92). Precision reached 93.8%, compared to 83.6% for YOLOv8 and 84.1% for HRFormer. Despite being slightly slower than YOLOv8 (18 FPS vs 45 FPS), the proposed model still meets real-time requirements and delivers significantly higher accuracy. These results suggest a well-balanced trade-off between speed and accuracy, with clear superiority in anatomical alignment and model generalization.

These results underscore that while high-speed detectors like YOLOv8 may be suitable for general object detection, their precision is insufficient for delicate clinical tasks such as acupoint localization. Our model’s structure-guided design ensures not only numerical superiority but also anatomical plausibility, essential for safe acupuncture guidance or medical navigation.

### 3.2 Robustness under obese body morphologies

**FIGURE 5:**
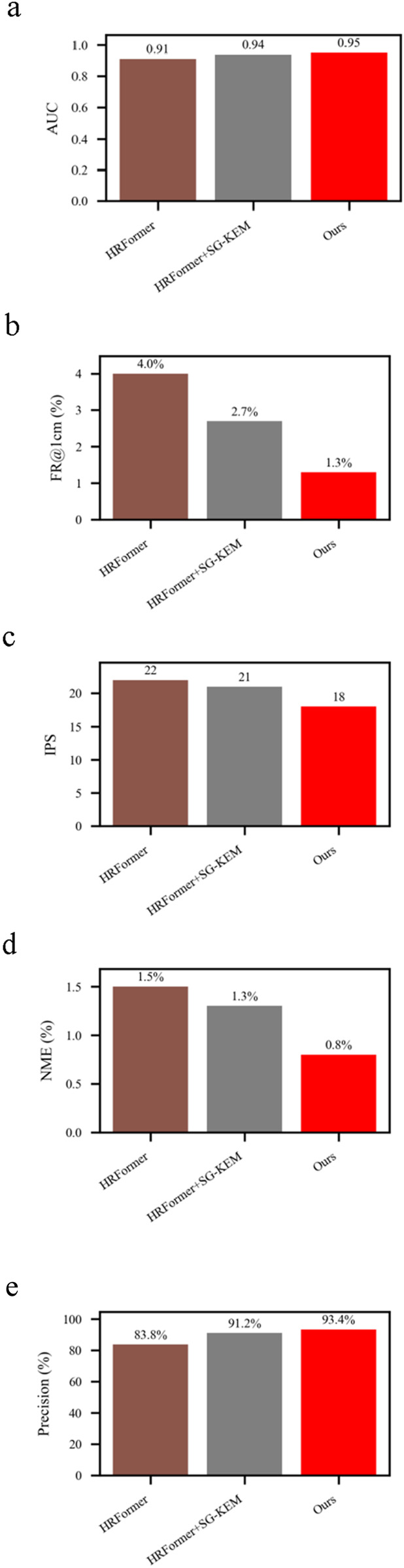
Results of ablation study under the obese subset. **(a)** Comparison of different models in AUC. **(b)** Comparison of different models in FR@1 cm (%). **(c)** Comparison of different models in IPS. **(d)** Comparison of different models in NME (%). **(e)** Comparison of different models in Precision (%).

To assess the robustness of our model in the presence of anatomical variations, we evaluated performance on a subset of obese subjects, whose back contours exhibit significant curvature, skin folds, and less-defined anatomical landmarks (Figure 5).reports the results of an ablation study under these challenging conditions.

We compared three variants:

HRFormer baseline, HRFormer + SG-KEM module and Full model (HRFormer + SG-KEM + structure-constrained loss).

NME decreased from 1.5% to 0.8%, representing a 46.7% improvement, while FR@1 cm dropped from 4.0% to 1.3%, confirming improved localization accuracy in anatomically complex regions. The addition of SG-KEM alone already improved NME to 1.3%, indicating that multi-scale structural priors provide benefit even without the loss constraint.

AUC improved from 0.91 to 0.95, and Precision increased from 83.8% to 93.4%, supporting the effectiveness of structure-constrained learning in handling size-induced anatomical distortion. FPS declined modestly from 22 to 18, but the real-time capability remained acceptable. These results indicate that our structure-guided model is well-adapted to variations in body morphology, enhancing its clinical applicability for diverse patient groups.

The improvements in obese individuals are particularly significant from a clinical perspective. In practice, acupoint palpation in overweight patients is more difficult due to tissue coverage and ambiguous bone landmarks. By leveraging a structure-constrained spatial normalization (TCM bone-measuring system), our model achieves robust predictions across body types. This is critical for real-world deployment in diverse populations, such as in hospitals or mobile healthcare units.

### 3.3 Generalization under illumination variation

**FIGURE 6:**
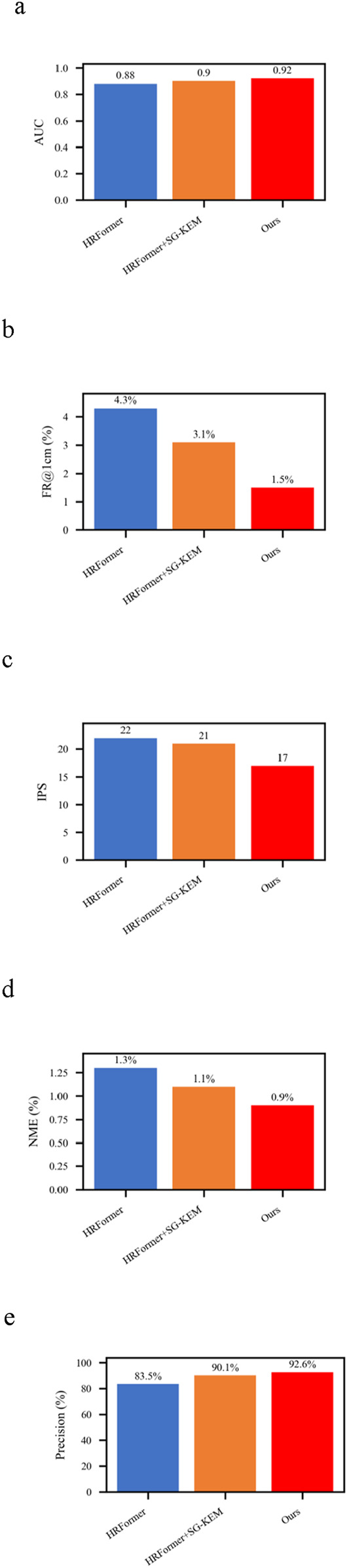
Results of ablation study under illumination variation. **(a)** Comparison of different models in AUC. **(b)** Comparison of different models in FR@1 cm (%). **(c)** Comparison of different models in IPS. **(d)** Comparison of different models in NME (%). **(e)** Comparison of different models in Precision (%).

Lighting conditions significantly impact image-based recognition systems, particularly in clinical settings where consistent lighting is hard to maintain. To evaluate generalization under such visual disturbances, we applied the same three model variants (HRFormer, HRFormer+SG-KEM, full model) to a test set modified with varying brightness and contrast levels. Results are summarized in (Figure 6).

The NME reduced from 1.3% in HRFormer to 0.9% in the full model, a 30.8% improvement, while the FR@1 cm dropped from 4.3% to 1.5%. These findings show the proposed model’s resilience in retaining spatial accuracy under degraded visual conditions.

AUC increased from 0.88 to 0.92, and Precision improved from 83.5% to 92.6%, confirming that both SG-KEM and structure-aware supervision enhance the model’s lighting invariance. Although FPS slightly decreased from 22 to 17, the model maintained real-time performance. These outcomes demonstrate that anatomical constraints improve illumination robustness, making the model suitable for dynamic clinical environments where lighting conditions may be inconsistent.

These results highlight that illumination invariance is not solely a function of network depth or capacity, but greatly benefits from domain-specific anatomical priors. The model’s integration of bone-referenced geometry enables it to rely less on pixel intensity and more on structural layout, a desirable trait in noisy or uncontrolled environments. This is particularly relevant for real-world TCM diagnosis settings using mobile devices or home-care robotics.

### 3.4 Summarize

Across all test conditions—standard, obese, and illumination-perturbed—the proposed model consistently demonstrates superior accuracy, robustness, and clinical relevance. The improvements observed in (Figures 4–6) stem from carefully designed modules that bridge anatomical priors with deep feature extraction. This structure-aware strategy enables robust acupoint localization suitable for complex and variable clinical environments.

## 4 Discussion

This study proposes a structure-aware acupoint localization framework that effectively integrates Traditional Chinese Medicine (TCM) principles with advanced deep learning techniques (Gang et al., 2011). By incorporating a high-resolution transformer backbone (HRFormer), a Structure-Guided Keypoint Estimation Module (SG-KEM), and a structure-constrained loss based on the bone-measuring method, the model achieves accurate and anatomically coherent localization of back acupoints.

The integration of SG-KEM significantly enhances spatial feature representation by guiding the model to focus on physiologically meaningful regions. This module leverages skeletal priors—such as scapular and spinal landmarks—that remain relatively stable across individuals, enabling the model to localize acupoints accurately even in anatomically ambiguous or low-contrast regions (Riegler et al., 2015). Furthermore, the structure-constrained loss enforces consistency in relative acupoint spacing based on TCM-defined proportions, enhancing physiological plausibility and improving generalization across different body types.

Experimental results confirm the effectiveness of the proposed approach. On a dataset comprising 430 back images with 19 annotated acupoints, the model achieved a normalized mean error (NME) of 0.6%, a failure rate (FR@1 cm) of 1.2%, and an AUC of 0.97. These outcomes reflect substantial gains in both spatial precision and anatomical consistency compared to multiple baselines, including HRFormer (Yuan Y. et al., 2021) alone, ViT (Yuan L. et al., 2021), and YOLOv8 (Sohan et al., 2024). Importantly, the model maintained real-time inference capability (18 IPS), meeting the demands of clinical deployment scenarios such as intelligent diagnosis, robot-assisted acupuncture, and posture-adaptive therapy systems (Cheng et al., 2024).

Ablation studies demonstrate the individual contributions of each component. The SG-KEM module reduced NME by 33.3%, while the addition of the structure-constrained loss further improved anatomical compliance and lowered NME to 0.6%.The benefits of this architecture extended to challenging clinical conditions. Under the obese subset (Mao et al., 2021), the proposed method achieved a 46.7% relative reduction in NME compared to HRFormer (from 1.5% to 0.8%), while improving precision from 83.8% to 93.4%. Similarly, under illumination variation, the model’s NME decreased from 1.3% to 0.9%, and precision increased from 83.5% to 92.6%, outperforming both the backbone and intermediate variants. These results substantiate the model’s robustness to patient morphology, lighting inconsistencies, and anatomical complexity, underscoring its viability for real-world clinical applications where such variability is common. This modular design supports flexible adaptation to various clinical applications and suggests potential for extension to other TCM body regions. These results substantiate the model’s robustness to patient morphology, lighting inconsistencies, and anatomical complexity, underscoring its viability for real-world clinical applications where such variability is common. In particular, the framework achieved consistent accuracy in images of obese individuals, where back curvature and surface texture differ significantly from standard anatomical presentations. These findings highlight the model’s resilience to intra-population variability and its suitability for broader clinical use.

Moreover, the proposed framework exemplifies how TCM domain knowledge can be systematically encoded into modern AI systems to enhance interpretability and trustworthiness. By embedding fixed anatomical constraints within both the network structure and training objective, the model yields acupoint predictions that are not only numerically accurate but also aligned with clinical and diagnostic expectations. This structure-aware paradigm offers a valuable reference for future development of AI systems that bridge traditional medical expertise with data-driven methodologies.

These findings not only confirm the technical robustness of the model but also support its alignment with classical meridian theories in Traditional Chinese Medicine (Yang et al., 2011). The accurate mapping of acupoints in proportionally normalized anatomical space resonates with the TCM concept of “bone-based measurement”, bridging empirical knowledge with data-driven precision. This provides a valuable foundation for modernizing diagnostic protocols and ensuring consistency in acupuncture-based interventions across practitioners and institutions.

While the current framework demonstrates robust performance on a curated dataset of back images, its application to dynamic scenarios—such as real-time tracking during respiration or movement—remains to be explored. Additionally, future work may incorporate multimodal sensing, including depth, thermal, or surface EMG signals, to further enhance performance under occlusion, poor lighting, or patient movement. Deployment optimization through model compression techniques may also broaden accessibility to portable and embedded hardware platforms (Pan et al., 2024; Dantas et al., 2024).

In summary, this study presents a clinically viable, structurally grounded, and computationally efficient solution for back acupoint localization. It underscores the potential of combining TCM anatomical principles with high-resolution deep learning to advance intelligent diagnosis and personalized treatment in integrative medicine.

## 5 Conclusion

In this study, we developed a novel acupoint localization framework that effectively integrates Traditional Chinese Medicine (TCM) anatomical knowledge with high-resolution deep learning techniques. By embedding the bone-measuring method into both the feature extraction and optimization stages, the proposed model achieves accurate, anatomically consistent localization of back acupoints—a task traditionally hindered by the back’s flat morphology and sparse visual landmarks.

Our approach combines the HRFormer backbone with a Structure-Guided Keypoint Estimation Module (SG-KEM) and a structure-constrained loss function rooted in TCM’s proportional anatomy. This design enables the model to capture spatially meaningful features and maintain physiologically plausible acupoint arrangements across individuals with varying body types. Experimental results demonstrate excellent localization performance (NME: 0.6%, FR@1 cm: 1.2%, AUC: 0.97), robust generalization under challenging conditions such as obesity and illumination variation, and real-time inference capability (18 IPS), confirming the model’s potential for clinical deployment. Importantly, ablation studies further revealed the model’s strong generalization under challenging conditions. Under the obese subset, the framework reduced NME from 1.5% to 0.8% and improved precision from 83.8% to 93.4%. In illumination variation scenarios, it achieved a 30.8% relative NME reduction (from 1.3% to 0.9%) and maintained high precision at 92.6%. These results confirm the model’s robustness across diverse body shapes and imaging environments, which are commonly encountered in real-world clinical settings.

Beyond technical contributions, this work exemplifies a promising direction in AI-assisted integrative medicine: translating traditional anatomical systems into machine-understandable priors. The framework offers a scalable and interpretable foundation for intelligent acupuncture navigation, standardized treatment planning, and TCM digitization. In the broader context of TCM, our method lays the groundwork for standardizing acupoint localization in clinical acupuncture, facilitating the integration of AI into meridian-based therapies. Furthermore, by quantifying traditionally qualitative anatomical concepts, it contributes to the digital transformation of acupuncture education, robot-assisted therapy, and international standard formulation. Future research will focus on expanding the dataset to include dynamic scenarios and diverse populations, incorporating multimodal imaging, and optimizing the model for deployment on portable diagnostic or therapeutic devices.

## Data Availability

The datasets [DMD-BAK] for this study can be found in the [https://www.kaggle.com/datasets/chunzheye/dmd-bak].
