# Supplementary material for: Structure-guided deep learning for back acupoint localization via bone-measuring constraints
Source: Front Physiol. 2025 Aug 26;16:1662104. doi: 10.3389/fphys.2025.1662104 (PMC12417426; doi:10.3389/fphys.2025.1662104)
Supplement: Supplementary file 1 [file Supplementaryfile1.docx]

Supplementary Material

# Supplementary Tables

**Table 1.** The correspondence between the code numbers and the acupoints.

| **Seq.** | **Type** | **Code** | **Acupoint** | **Index** |
| --- | --- | --- | --- | --- |
| 1 | DU | DU14 | Dazhui | 1 |
| 2  3  4  5  6  7  8  9  10  11  12  13  14  15 | BL  BL  BL  BL  BL  BL  BL  BL  BL  BL  BL  BL  BL  BL | BL11  BL12  BL13  BL14  BL15  BL17  BL18  BL19  BL20  BL21  BL22  BL23  BL25  BL43 | Dazhu  Fengmen  Feishu  Jueyinshu  Xinshu  Geshu  Ganshu  Danshu  Pishu  Weishu  Sanjiaoshu  Shenshu  Dachangshu  Gaohuang | 2/3  4/5  6/7  8/9  10/11  12/13  14/15  16/17  18/19  20/21  22/23  24/25  26/27  28/29 |
| 16 | GB | GB21 | Jianjing | 30/31 |
| 17  18  19 | SI  SI  SI | SI9  SI10  SI11 | Jianzhen  Naoshu  Tianzong | 32/33  34/35  36/37 |

**Table 2.** Acupoint-skeletal correlations with topologic descriptors.

| **Seq.** | **Acupoint** | **Skeletal Landmark** | **Topological Relationship** |
| --- | --- | --- | --- |
| 1 | Dazhui | C7 Spinous Process | Below C7 (midline) |
| 2  3  4  5  6  7  8  9  10  11  12  13  14  15 | Dazhu  Fengmen  Feishu  Jueyinshu  Xinshu  Geshu  Ganshu  Danshu  Pishu  Weishu  Sanjiaoshu  Shenshu  Dachangshu  Gaohuang | T1 Spinous Process  T2 Spinous Process  T3 Spinous Process  T4 Spinous Process  T5 Spinous Process  T7 Spinous Process  T9 Spinous Process  T10 Spinous Process  T11 Spinous Process  T12 Spinous Process  L1 Spinous Process  L2 Spinous Process  L4 Spinous Process  T4 Spinous Process | Below T1, 1.5 cun lateral  Below T2, 1.5 cun lateral  Below T3, 1.5 cun lateral  Below T4, 1.5 cun lateral  Below T5, 1.5 cun lateral  Below T7, 1.5 cun lateral (scapula level)  Below T9, 1.5 cun lateral  Below T10, 1.5 cun lateral  Below T11, 1.5 cun lateral  Below T12, 1.5 cun lateral  Below L1, 1.5 cun lateral  Below L2, 1.5 cun lateral (umbilicus level)  Below L4, 1.5 cun lateral (iliac crest level)  Below T4, 3 cun lateral (medial scapula) |
| 16 | Jianjing | C7 and Acromion | Midpoint of C7-Acromion line |
| 17  18  19 | Jianzhen  Naoshu  Tianzong | Posterior Axillary Fold  Scapular Spine  Scapular Spine | 1 cun above axillary fold  Inferior to scapular spine  Center of infraspinous fossa |

**Table 3.** Data Augmentation Strategies

| **Augmentation Type** | **Description** | **Parameter Range/Probability** |
| --- | --- | --- |
| Random Horizontal Flip | Symmetric left-right flip of the image | Probability = 0.5 |
| Random Rotation | Rotation operation in affine transformation | Angle ∈ [−15°, +15°] |
| Random Scaling | Affine scaling operation with the center unchanged | Scaling ratio ∈ [0.9, 1.1] |
| Random Brightness Perturbation | Adjust the overall brightness of the image | Adjustment factor ∈ [0.8, 1.2] |
| Random Contrast Perturbation | Change the contrast of the image | Adjustment factor ∈ [0.8, 1.2] |

**Table 4.** Parameters of four stages of the HRFormer.

| **Stage** | **Number of Branches** | **Resolution Ratio** | **Number of Transformer Blocks per Branch** | **Embedding Dimension per Layer（C）** |
| --- | --- | --- | --- | --- |
| 1 | 1 | 1/4 | 2 | 64 |
| 2 | 2 | 1/4，1/8 | 2，2 | 64，128 |
| 3 | 3 | 1/4 ，1/8，1/16 | 2，2，2 | 64，128，256 |
| 4 | 4 | 1/4，1/8，1/16，1/32 | 2，2，2，2 | 64，128，256，512 |
