# Supplementary material for: Structure-guided deep learning for back acupoint localization via bone-measuring constraints
Source: Front Physiol. 2025 Aug 26;16:1662104. doi: 10.3389/fphys.2025.1662104 (PMC12417426; doi:10.3389/fphys.2025.1662104)

Supplementary Material

# Supplementary Figure

**Figure 1.** Structure of HRFormer.

a:The HRFormer block is composed of local-window self-attentionm and feed-forward network (FFN) with depth-wise convolution


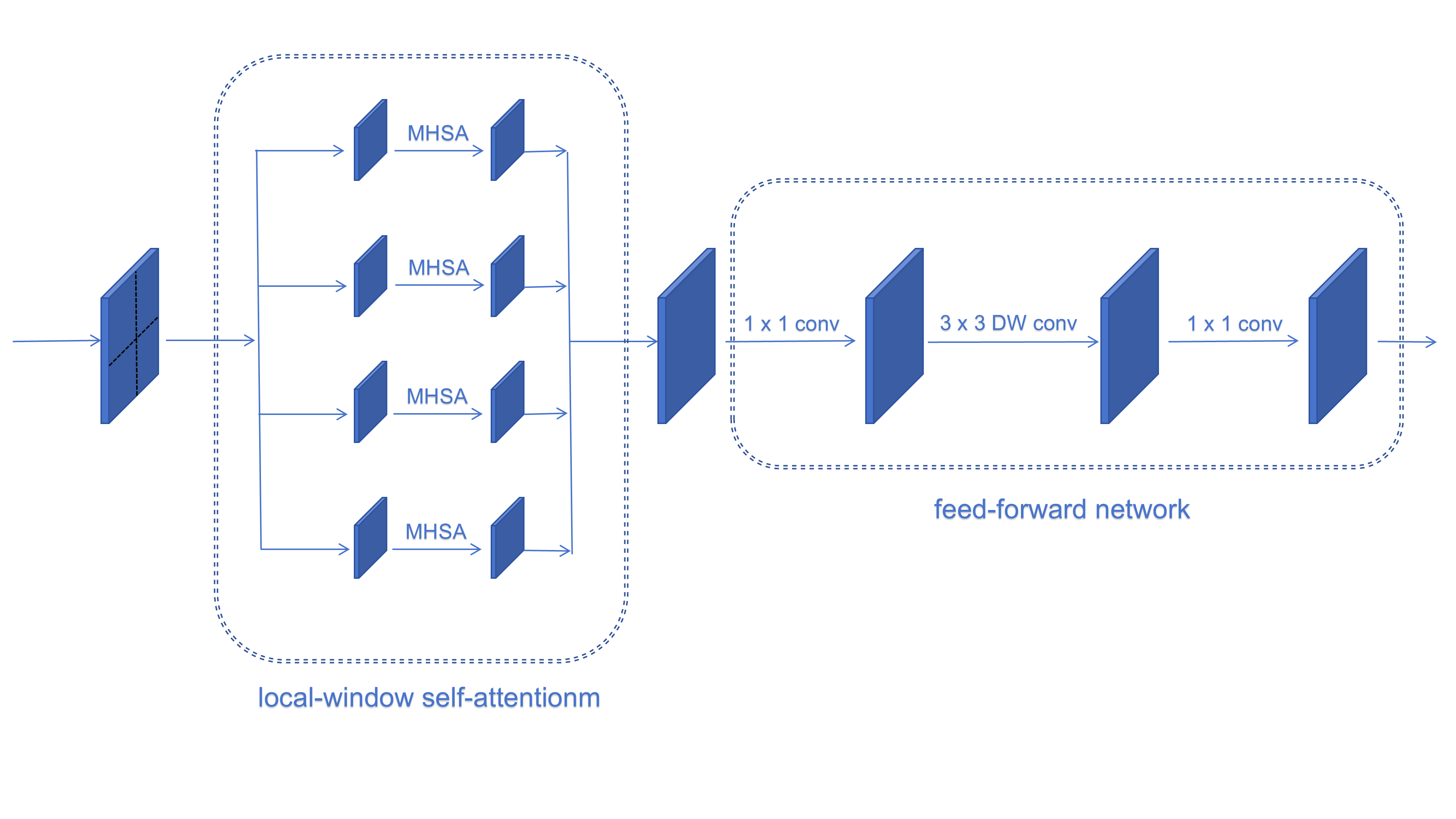


b:Illustrating the HRFormer architecture.


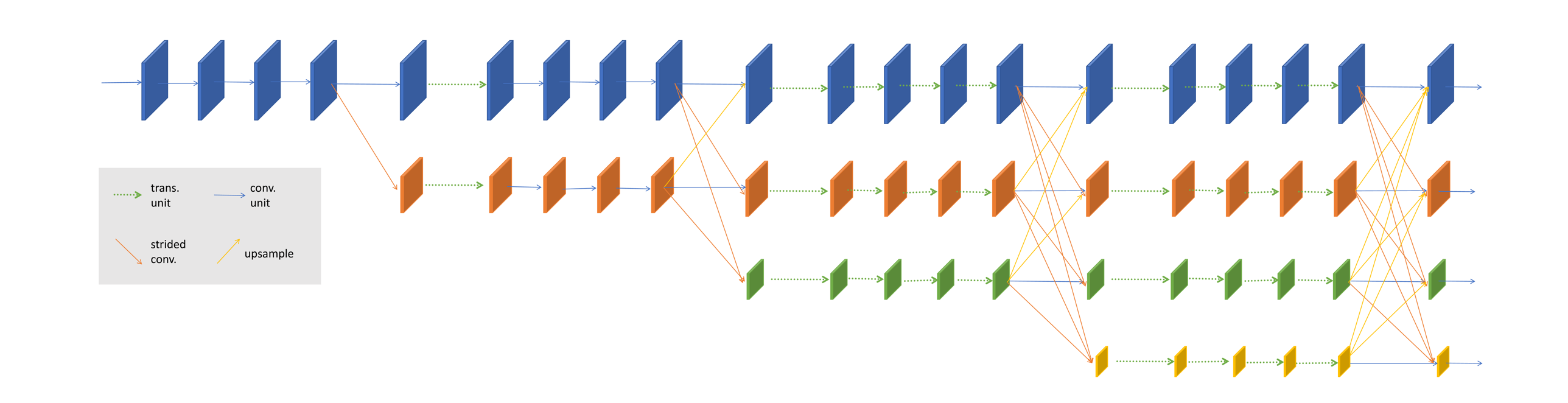


**Figure 2.** Structure of SG-KEM Module.


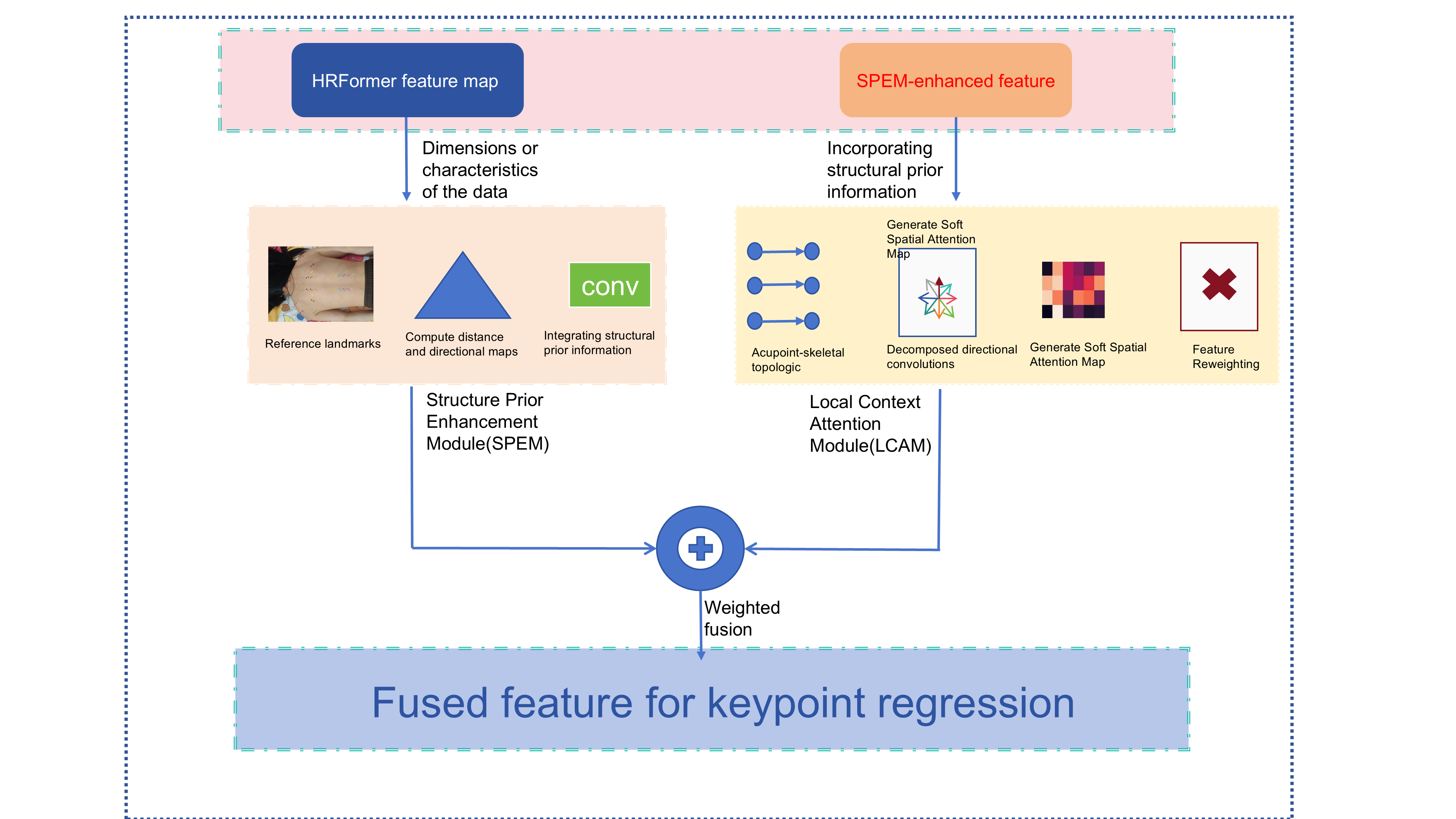


**Figure 3.** Structure-Constrained Losses Function.


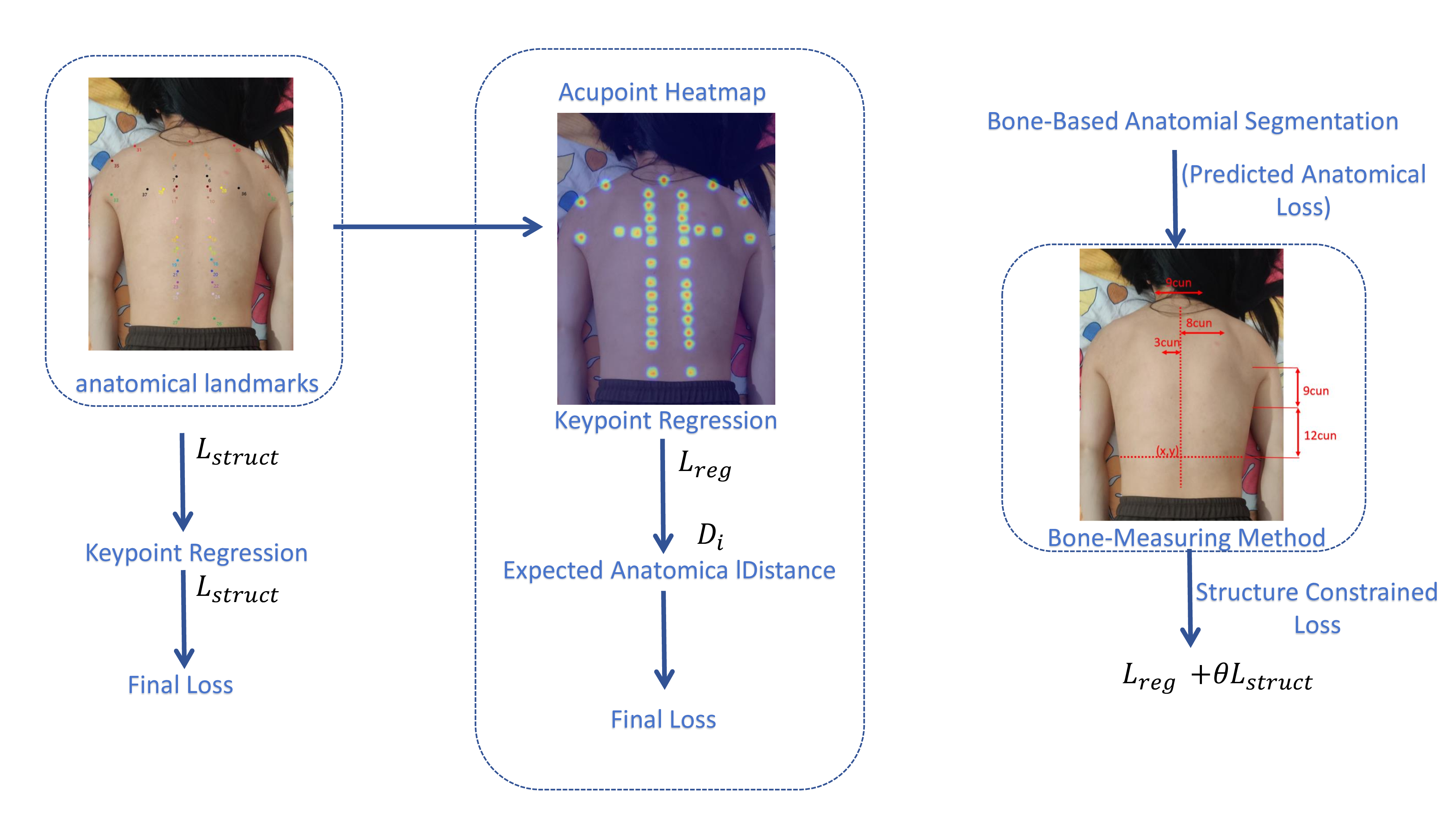


**Figure 4.** Performance comparison of different models .

a:Comparison of different models in AUC


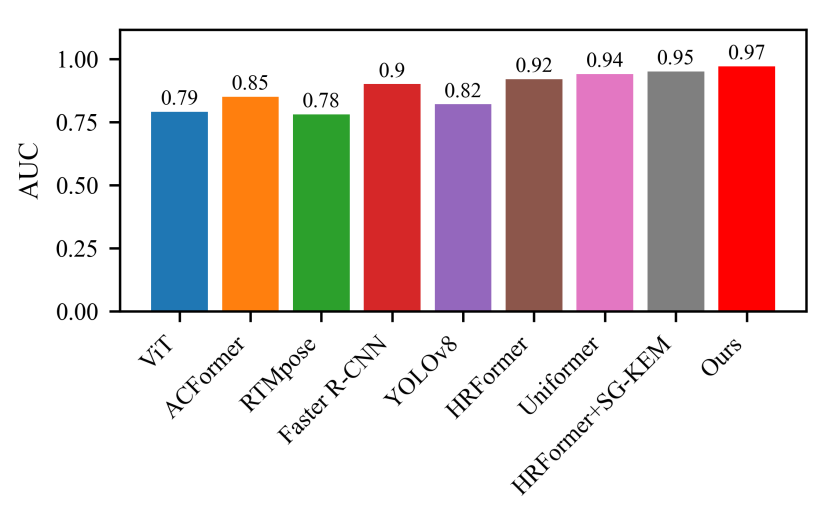


b:Comparison of different models in FR@1cm(%).

**
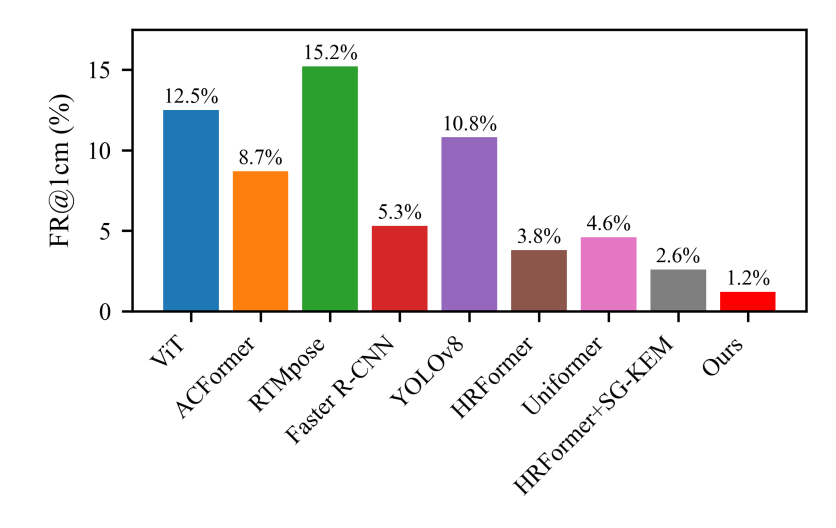
**

c:Comparison of different models in IPS.


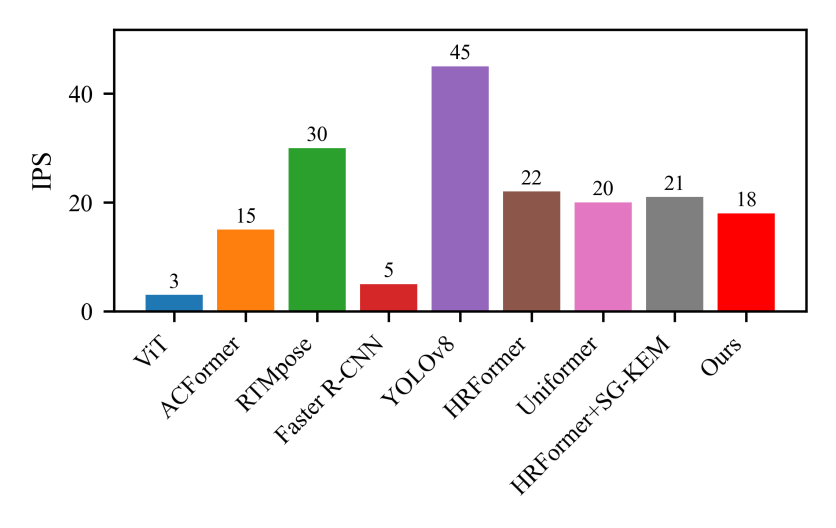


d:Comparison of different models in NME(%).


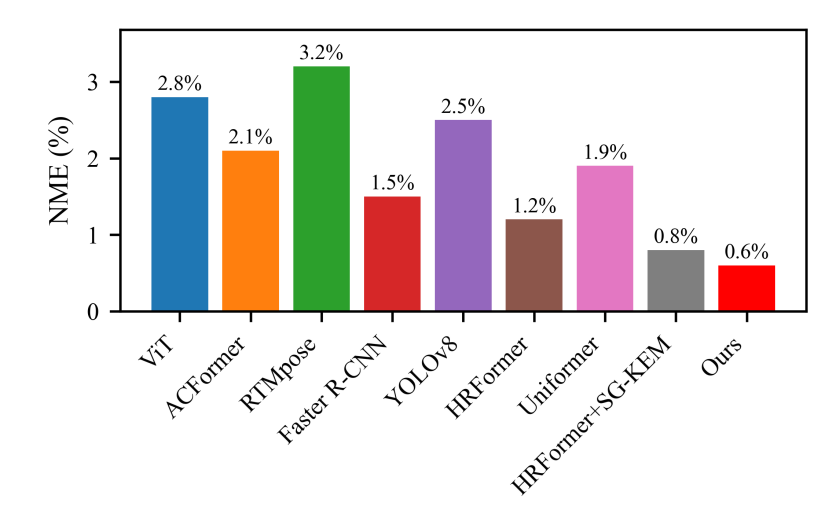


E:Comparison of different models in Precision(%).


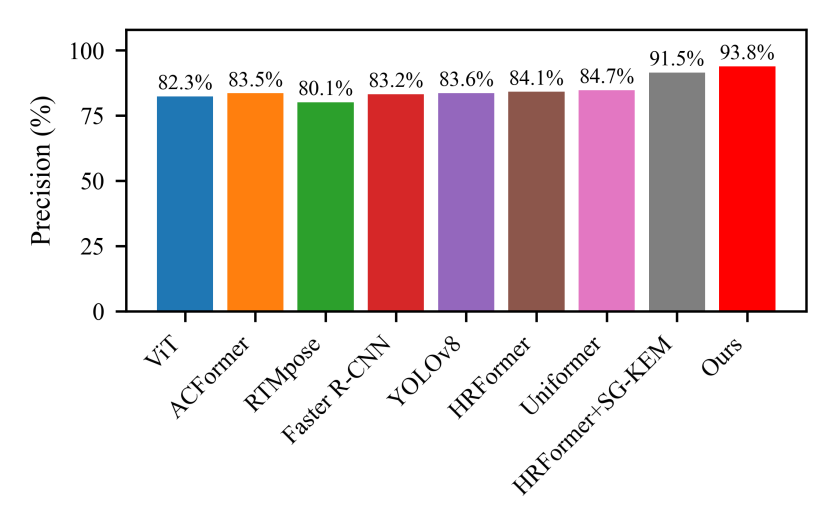


**Figure 5.** Results of ablation study under the obese subset.

a:Comparison of different models in AUC

**
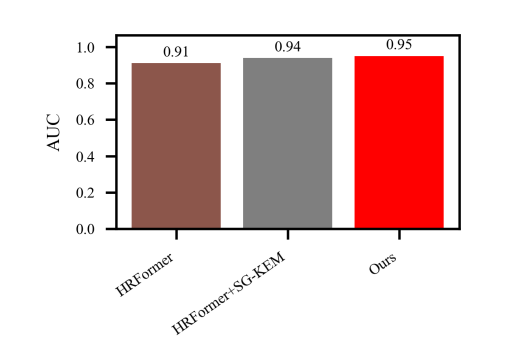
**

b:Comparison of different models in FR@1cm(%).


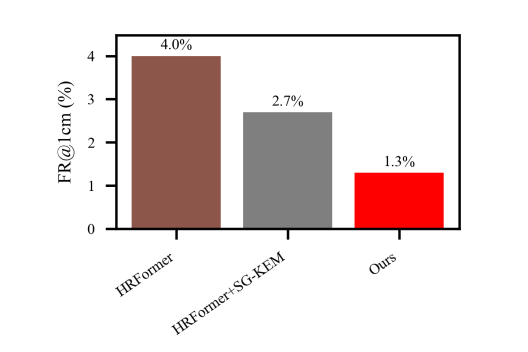


c:Comparison of different models in IPS.


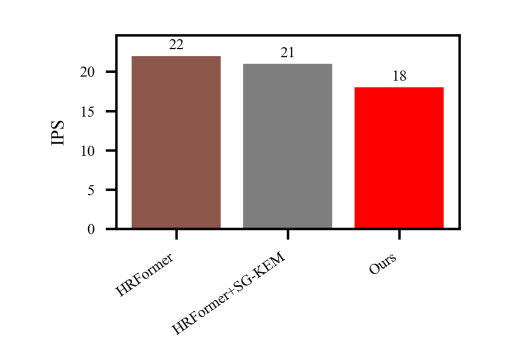


d:Comparison of different models in NME(%).


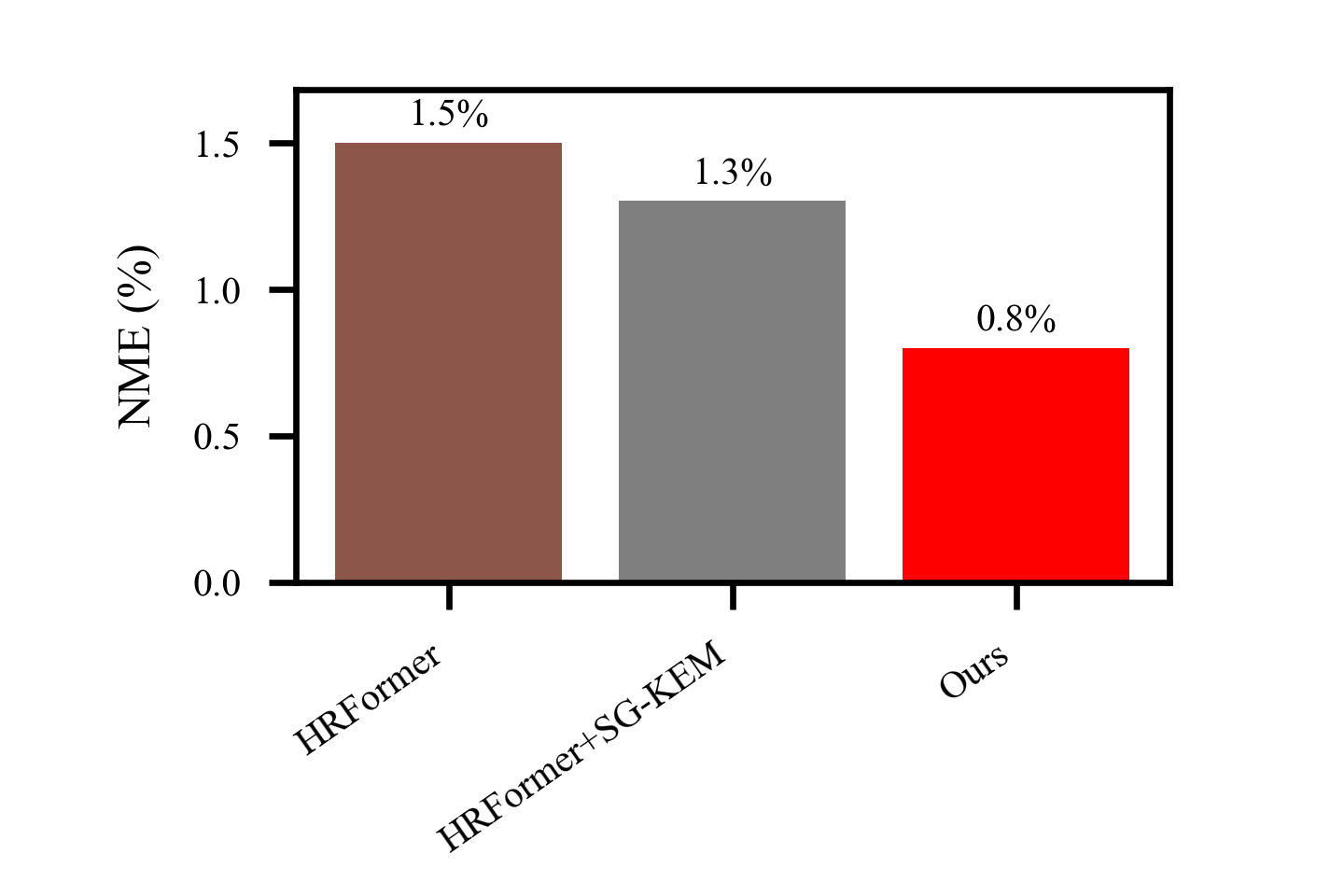


E:Comparison of different models in Precision(%).


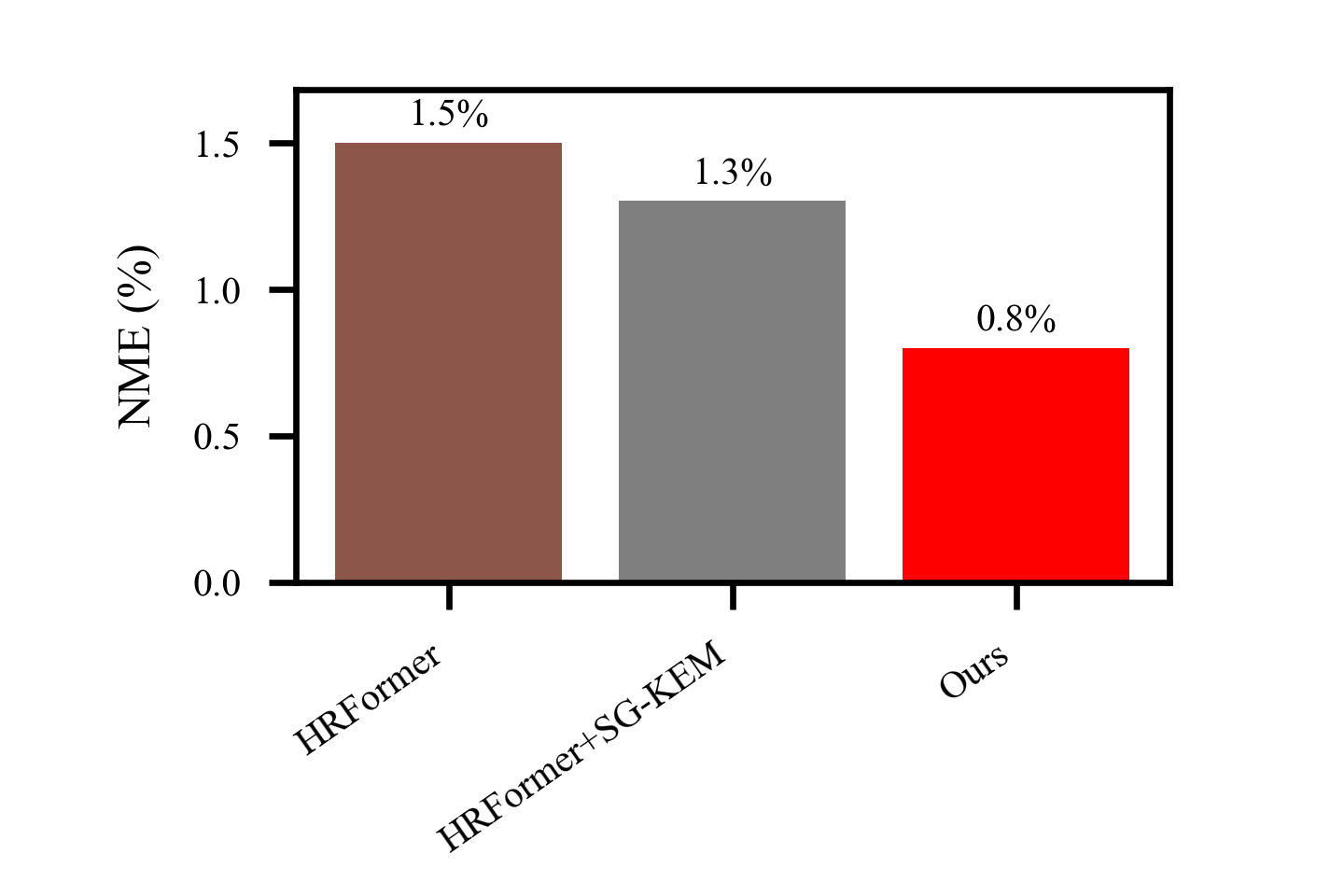


**Figure 6.** Results of ablation study under illumination variation.

a:Comparison of different models in AUC

**
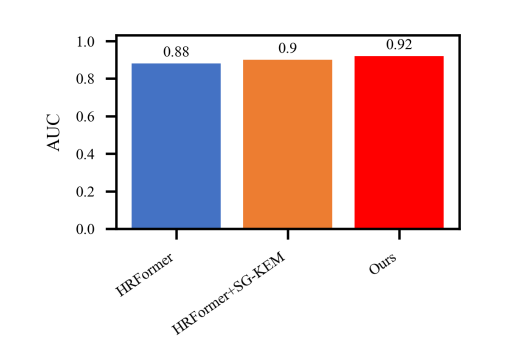
**

b:Comparison of different models in FR@1cm(%).


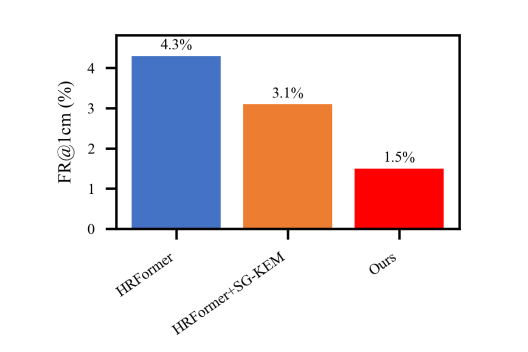


c:Comparison of different models in IPS.


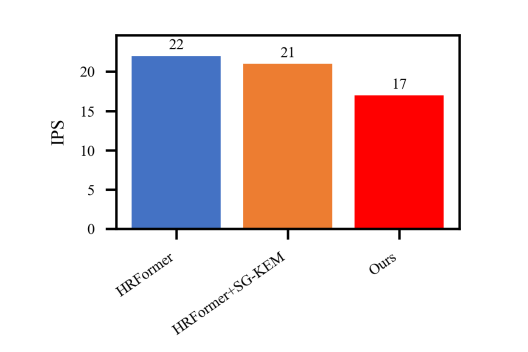


d:Comparison of different models in NME(%).


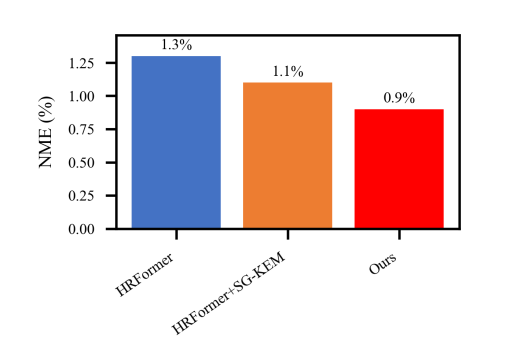


E:Comparison of different models in Precision(%).


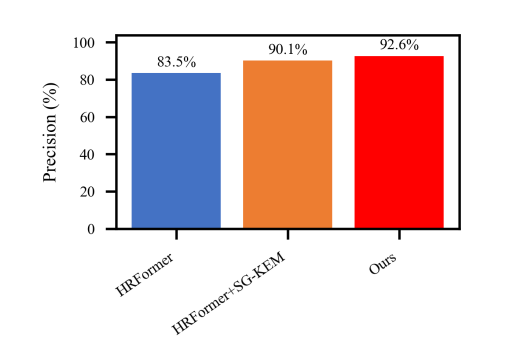

Supplement: Supplementary file 2 [file Supplementaryfile2.docx]
